# A latent profile analysis of financial toxicity and its association with anxiety and depression in patients with hematologic malignancies

**DOI:** 10.1016/j.apjon.2025.100761

**Published:** 2025-07-22

**Authors:** Guiyuan Ma, Nannan Long, Ping Mao, Yuanyuan Li, Fang Li, Chengyuan Li

**Affiliations:** a Nursing Department, The Third Xiangya Hospital, Central South University, Changsha, China; b Xiangya School of Nursing, Central South University, Changsha, China; c Hematology Department, The Third Xiangya Hospital, Central South University, Changsha, China; d Lymphoma Hematology Department, Hunan Cancer Hospital, Changsha, China; e Hematology Department, Hunan Provincial People's Hospital, Changsha, China

**Keywords:** Hematological malignancies, Financial toxicity, Latent profile analysis, Anxiety, Depression

## Abstract

**Objective:**

To identify latent subgroups of financial toxicity among patients with hematologic malignancies, examine associated sociodemographic and clinical predictors, and explore the relationship between financial toxicity and anxiety-depression symptoms.

**Methods:**

A cross-sectional study was conducted among 486 patients with hematologic malignancies recruited through convenience sampling from provincial-level hospitals in Hunan Province, China. Participants completed the General Information Questionnaire, the Patient-Reported Outcome Financial Toxicity (PRO-FT) Scale, the Patient Health Questionnaire-4 (PHQ-4), and the Perceived Social Support Scale (PSSS). Latent profile analysis (LPA) was used to identify subgroups of financial toxicity. Multinomial logistic regression was employed to explore influencing factors, and Kruskal–Wallis tests were used to compare anxiety and depression levels across the identified subgroups.

**Results:**

Three distinct profiles of financial toxicity were identified: “No financial toxicity” (13.99%), “Mild financial toxicity” (38.68%), and “Moderate financial toxicity” (47.33%). Compared with the no-toxicity profile, predictors of being in the mild or moderate toxicity profiles included sex, marital status, occupation, residence, family monthly income, treatment stage, insurance, and social support. When the mild toxicity profile was used as the reference, progression to the moderate profile was further associated with residence, family monthly income, diagnosis, chronic disease, course of disease, insurance, and social support. Higher levels of financial toxicity were significantly associated with increased anxiety and depression scores.

**Conclusions:**

Financial toxicity among patients with hematologic malignancies is heterogeneous and closely linked to sociodemographic and clinical characteristics. Moreover, increased financial toxicity is associated with elevated psychological distress. Target interventions addressing financial strain and enhancing psychological support are essential to improve patient outcomes.

## Introduction

Hematologic malignancies (HMs) have demonstrated a steadily increasing global incidence in recent decades.^1^ According to the latest Global Cancer Statistics 2022 report released by the International Agency for Research on Cancer (IARC) in April 2024,^2^ non-Hodgkin lymphoma and leukemia collectively rank as the tenth most prevalent cancers worldwide, with substantial associated mortality rates. HMs are a group of malignant diseases characterized by abnormal proliferation and differentiation of blood cells and disruption of normal hematopoietic mechanisms.^2^ They include leukemia, multiple myeloma, lymphoma, and myelodysplastic syndromes.^1^ With the development of precision medicine and molecularly targeted therapy, significant progress has been made in the treatment of hematological tumors. However, patients with HMs incur substantial long-term healthcare expenditures due to the characteristic features of prolonged treatment duration, high costs of therapeutic regimens, and elevated relapse rates.^3^ For instance, in leukemia management, conventional chemotherapy incurs annual costs ranging from ¥50,000 to ¥150,000, whereas targeted therapies escalate expenses to ¥300,000–500,000 annually.^3^^,^^4^ Hematopoietic stem cell transplantation, a critical intervention for eligible patients, typically requires a single-treatment cost exceeding ¥500,000–1,000,000. Similarly, multiple myeloma treatment costs approximately ¥200,000–600,000 annually, with combination therapies incorporating Chimeric Antigen Receptor T-cell (CAR-T) immunotherapy potentially exceeding ¥1,000,000 per cycle.^5^ Furthermore, long-term follow-up assessments, supportive care, and complication management further increase healthcare expenditures.^4^ These treatment costs impose not only a significant objective financial burden on patients' families but may also precipitate psychological distress, such as anxiety and depression, arising from cost-related concerns.^6^ This dual impact of objective economic strain and subjective financial distress, induced by disease-related medical expenses, is termed financial toxicity.^6^

Most existing studies on financial toxicity assess it using total or mean scores from tools such as the Comprehensive Score for Financial Toxicity (COST),^7^^,^^8^ assuming a linear and uniform impact across different cancer populations.^9^^,^^10^ This variable-centered, unidimensional approach overlooks individual differences and fails to identify high-risk subgroups, particularly among patients with HMs who often experience complex disease trajectories and substantial treatment burdens.^1^ Unlike solid tumors, HMs frequently involve prolonged and unpredictable treatment processes, such as multiple rounds of chemotherapy, hematopoietic stem cell transplantation, and long-term follow-up, which can lead to high and fluctuating out-of-pocket costs.^1^^,^^11^^,^^12^ Combined with inconsistent insurance reimbursement policies, these factors contribute to significant heterogeneity in financial distress. In recent years, Latent Profile Analysis (LPA)^13^ has emerged as a person-centered method increasingly applied in health behavior and psychological research. It enables the identification of unobserved subgroups based on patterns of responses across continuous indicators, offering a more nuanced understanding of financial toxicity. Although some studies^13^^,^^14^ have used LPA to classify cancer survivors into high-, moderate-, and low-risk financial toxicity groups among elderly survivors or young/middle-aged cancer patients, these analyses often combine various cancer types without accounting for disease-specific differences in treatment and cost burden. Given the unique financial challenges faced by patients with HMs,^1^^,^^12^ applying LPA in this context may improve the identification of vulnerable subgroups and provide a methodological foundation for targeted economic interventions.

The influencing factors of financial toxicity span multiple factors across individual, family, and societal levels. At the individual level, these include age,^15^ race,^15^ educational level,^14^ and more. At the family level, factors such as monthly family income^16^ and insurance type^11^ are involved. Additionally, societal-level factors like social support may also affect level of financial toxicity. Multiple evidence indicate that the level of social support determines the financial toxicity in cancer patients, such as Li's study showed that non-Hodgkin lymphoma patients with insufficient social support may experience severe financial toxicity.^9^ Social support reduces patients' economic burden and psychological stress through forms such as direct financial assistance, indirect cost control, and information support networks, thereby effectively mitigating the level of financial toxicity.^9^^,^^10^^,^^17^ However, these influencing factors have not been explored across different heterogeneous patient subgroups. Meanwhile, studies have shown that financial toxicity is an important predictor of mental health impairment in cancer patients,^18^ especially increasing the risk of negative emotions such as anxiety and depression. This stems from financial stress and a vicious cycle of limited treatment options, poor efficacy expectations, reduced quality of life, and emotional distress.^19^^,^^20^ However, there is an obvious limitation in the existing studies: most of them focus on the overall impact of financial toxicity as a homogenized risk factor and ignore the possible differences in psychological status among patients with different subtypes of financial toxicity.^20^ This cognitive gap not only restricts our systematic understanding of the economic-psychological double burden of patients with HMs, but also makes it challenging to formulate stepwise and precise psychosocial support programs for patient groups with different risk characteristics, which ultimately affects the actual effects of interventions.^19^

Therefore, this study aims to identify distinct profiles and associated characteristics of financial toxicity in HMs patients using LPA, and investigate their relationships with anxiety-depressive symptoms. These findings will provide a theoretical foundation for developing targeted interventions to mitigate financial toxicity in HMs population.

## Methods

### Sample

This cross-sectional study was conducted from October to December 2024 in three provincial-level hospitals in Hunan Province, China. A convenience sampling method was employed to recruit patients from hematology-oncology departments. Inclusion criteria were as follows: (1) pathologically confirmed diagnosis of a hematologic malignancy; (2) currently hospitalized; (3) adequate communication ability; and (4) full awareness of their diagnosis with voluntary participation and signed informed consent, for underage participants, informed consent was obtained from both the patients and their legal guardians prior to questionnaire completion. Exclusion criteria included: (1) cognitive impairment or psychiatric disorders; and (2) severe comorbidities affecting major organ systems (e.g., cardiovascular, cerebrovascular, or renal diseases).

To ensure sufficient statistical power for LPA and reliable identification of small subgroups, a minimum sample size of 300 was recommended.^21^ In this study, a total of 486 participants were included, which provided adequate power for the LPA.

### Measurement

#### General information questionnaire

A structured general information questionnaire was developed encompassing three hierarchical levels. (1) Individual level: age, sex, marital status, educational attainment, occupation, diagnosis, presence of chronic diseases, disease duration, treatment phase, relapse status, complications, and sense of coherence. (2) Family level: place of residence, monthly family income, and health insurance coverage. (3) Societal level: social support.

#### Financial toxicity

Financial toxicity was assessed using the COST scale, originally developed by de Souza et al.^7^ and translated into Chinese by Yu et al., in 2017.^8^ Validation studies among Chinese cancer patients demonstrated strong reliability and validity, with a Cronbach's alpha of 0.889, test-retest Pearson correlation coefficients ranging from 0.77 to 0.98, and content validity indices between 0.83 and 1.00. The COST scale comprises 11 items across three domains: active economic expenditure, passive economic resources, and psychosocial responses. Items are rated on a 5-point Likert scale from 0 (“not at all”) to 4 (“very much”). Items 1, 6, 7, and 11 are positively scored, while the remaining items are reverse-scored. The total score ranges from 0 to 44, with higher scores indicating lower financial toxicity. Score interpretation is as follows: > 26, no financial toxicity; 14–26, mild financial toxicity; 11–13, moderate financial toxicity; and ≤ 10, severe financial toxicity. In this study, the COST scale demonstrated excellent internal consistency with a Cronbach's alpha of 0.89.

#### Anxiety and depression

Anxiety and depression symptoms were evaluated using the Patient Health Questionnaire-4 (PHQ-4), a brief screening tool developed by Löwe et al.^22^ The PHQ-4 combines the first two items of the PHQ-9 (PHQ-2) to assess depressive symptoms and the first two items of the GAD-7 (GAD-2) to assess anxiety symptoms, reflecting patients' emotional status over the preceding two weeks. Each item is rated on a 4-point scale: 0 (“not at all”), 1 (“several days”), 2 (“more than half the days”), and 3 (“nearly every day”). Total scores range from 0 to 12, with 0–2 indicating no symptoms, 3–5 mild, 6–8 moderate, and 9–12 severe anxiety or depression. In this study, the PHQ-4 demonstrated good reliability with a Cronbach's alpha of 0.84.

#### Social support

Social support was measured using the Perceived Social Support Scale (PSSS), developed by Zimet et al., in 1990.^23^ The scale consists of 12 items that assess an individual's perceived level of support from various sources, including family, friends, and significant others. Each item is rated using a 7-point Likert scale ranging from 1 (strongly disagree) to 7 (strongly agree), yielding a total score between 12 and 84. Higher scores indicate greater perceived social support. Based on the total score, perceived social support is categorized as low (≤ 36), moderate (37–60), or high (61–84). In this study, the Cronbach's alpha coefficient for the scale was 0.90, indicating excellent internal consistency.

### Data collection and quality control

Following approval from the hospital's nursing department, data were collected from patients with hematological malignancies (HMs) by the principal researchers and two trained investigators who underwent standardized training. Prior to data collection, a unified protocol was used to explain the study's purpose, significance, content, and precautions to participants. All participants were instructed to complete the questionnaire independently and anonymously within the allotted time. For participants who experienced difficulties in completing the questionnaire due to literacy or comprehension challenges, trained investigators conducted oral interviews and recorded responses on their behalf. Upon completion, questionnaires were immediately reviewed for completeness and accuracy; any missing or ambiguous responses were clarified with the participant before final submission. A total of 500 questionnaires were distributed, with 492 returned (8 were not returned within the specified timeframe). Among these, 23 questionnaires were completed by investigators via oral interviews due to participants' limited literacy. Following double verification by two independent reviewers, invalid questionnaires were excluded (4 with patterned responses and 2 with completion times under 5 minutes), resulting in 486 valid questionnaires. The effective response rate was 98.78%.

### Statistical analysis

LPA was performed using Mplus version 8.0. The analysis was based on the 11 item scores from the Comprehensive Financial Toxicity Score (COST) scale reported by patients with hematologic malignancies. Models were sequentially estimated beginning with a one-class solution and increasing up to five classes. Model fit indices were compared to determine the optimal number of latent profiles, considering both statistical criteria and theoretical interpretability.

The following fit indices were used to evaluate model adequacy: (1) Information criteria: Akaike Information Criterion (AIC), Bayesian Information Criterion (BIC), and sample-size adjusted BIC (aBIC). Lower values indicate better model fit. (2) Classification accuracy: Entropy, ranging from 0 to 1, reflects classification precision, with values closer to 1 indicating better classification quality. (3) Likelihood ratio tests: The Lo–Mendell–Rubin adjusted likelihood ratio test (LMR) and Bootstrap Likelihood Ratio Test (BLRT) were used to compare adjacent models. A *P*-value < 0.05 suggests that the model with k classes fits significantly better than the model with k−1 classes. Although statistical indices guided model selection, interpretability and theoretical relevance of the classes were also considered in determining the final model.

All subsequent statistical analyses were conducted using SPSS version 26.0 (IBM Corp., Armonk, NY, USA). Continuous variables were summarized using mean ± standard deviation (SD) or median and interquartile range [M (Q1, Q3)], and categorical variables were presented as frequencies and percentages. Differences in demographic characteristics and scale scores across financial toxicity profiles were assessed using chi-square tests or Kruskal–Wallis rank sum tests, as appropriate. Variables showing statistically significant differences among financial toxicity profiles were further analyzed using Logistic regression to identify predictors of profile membership. Due to non-homogeneity of variance, the Kruskal–Wallis test was also applied to compare anxiety and depression scores across latent financial toxicity classes. A two-tailed *P*-value < 0.05 was considered statistically significant.

### Ethical considerations

This study was approved by the Institutional Review Board of the Third Xiangya Hospital, Central South University (Approval No: 2024-S425). Prior to enrollment, all participants provided written informed consent after receiving a detailed explanation of the study's objectives, procedures, potential risks, and anticipated benefits. Participants were informed of their right to withdraw from the study at any time without any impact on their clinical care or treatment. All data collected were anonymized using unique numerical identifiers and securely stored on a password-protected computer accessible only to the research team.

## Results

### General information

A total of 486 patients with HMs completed the survey. The majority of participants (41.36%) were aged between 40 and 59 years. Most were married (79.01%) and had attained at least a junior high school education (79.63%). Nearly half (42.59%) were employed as workers or farmers. Regarding family income, 38.48% reported a monthly household income between 5000 and 10,000 Yuan (USD 1 ≈ CNY 7.22). Detailed demographic characteristics are presented in Table 1.

**Table 1:** General data of patients with HMs in the three categories between groups.

Characteristics		Overall (*n* ​= ​486) *n* (%)	C1 (*n* ​= ​68) *n* (%)	C2 (*n* ​= ​188) *n* (%)	C3 (*n* ​= ​230) *n* (%)	Value	*P*-value
**Age (years)**	0–19	21(4.32)	2 (2.94)	14 (7.45)	5 (2.17)	51.816^a^	< 0.001
	20–39	93(19.14)	2 (2.94)	33 (17.55)	58 (25.22)		
	40–59	201 (41.36)	26 (38.24)	66 (35.11)	109 (47.39)		
	60–79	131 (26.95)	35 (51.47)	57 (30.32)	39(16.96)		
	80+	40 (8.23)	3 (4.41)	18 (9.57)	19 (8.26)		
**Sex**	Male	262 (53.91)	43 (63.24)	112 (59.57)	107 (46.52)	9.860^b^	0.007
	Female	224 (46.09)	25 (36.76)	76 (40.43)	123 (53.48)		
**Marital status**	Unmarried	62 (12.76)	3 (4.41)	30 (15.96)	29 (12.61)	42.346^a^	< 0.001
	Married	384 (79.01)	50 (73.53)	145 (77.13)	189 (82.17)		
	Divorced	21 (4.32)	1 (1.47)	11 (5.85)	9 (3.91)		
	Widowed	19 (3.91)	14 (20.59)	2 (1.06)	3 (1.30)		
**Educational level**	Primary school and below	99 (20.37)	11 (16.18)	31 (16.49)	57 (24.78)	20.857^b^	0.002
	Junior high school	167 (34.36)	14 (20.59)	63 (33.51)	90 (39.13)		
	Senior high school	94 (19.34)	16 (23.53)	39 (20.74)	39 (16.96)		
	College degree or above	126 (25.93)	27 (39.71)	55 (29.26)	44 (19.13)		
**Occupation**	Farmer/Worker	207 (42.59)	11 (16.18)	67 (35.64)	129 (56.09)	64.494^b^	< 0.001
	Enterprise personnel	69 (14.20)	6 (8.82)	28 (14.89)	35 (15.22)		
	Civil servant/public institution	43 (8.85)	17 (25.00)	18 (9.57)	8 (3.48)		
	Individual/Retired/Unemployed	167 (34.36)	34 (50.00)	75 (39.89)	58 (25.22)		
**Residence**	Rural	208 (42.80)	7 (10.29)	65 (34.57)	136 (59.13)	73.383^b^	< 0.001
	Township	74 (15.23)	7 (10.29)	34 (18.09)	33 (14.35)		
	City	204 (41.98)	54 (79.41)	89 (47.34)	61 (26.52)		
**Family monthly income (Yuan)**	> 10,000	140 (28.81)	2 (2.94)	41 (21.81)	97 (42.17)	102.605^b^	< 0.001
	5000-10,000	187 (38.48)	13 (19.12)	79 (42.02)	95 (41.30)		
	< 5000	159 (32.72)	53 (77.94)	68 (36.17)	38 (16.52)		
**Diagnosis**	Multiple myeloma	167 (34.36)	23 (33.82)	91 (48.40)	53 (23.04)	35.263^a^	< 0.001
	Lymphoma	139 (28.60)	23 (33.82)	38 (20.21)	78 (33.91)		
	Leukemia	164 (33.74)	21 (30.88)	50 (26.60)	93 (40.43)		
	Others	16 (3.29)	1 (1.47)	9 (4.79)	6 (2.61)		
**Chronic disease**	None	358 (73.66)	49 (72.06)	109 (57.98)	200 (86.96)	44.878^b^	< 0.001
	Yes (Hypertension, diabetes, coronary heart disease, chronic obstructive pulmonary disease)	128 (26.34)	19 (27.94)	79 (42.02)	30 (13.04)		
**Course of disease**	1–6 months	249 (51.23)	45 (66.18)	121 (64.36)	83 (36.09)	51.685^a^	< 0.001
	6–12 months	99 (20.37)	7 (10.29)	37 (19.68)	55 (23.91)		
	1–3 years	104 (21.40)	9 (13.24)	21 (11.17)	74 (32.17)		
	3+ years	34 (7.00)	7 (10.29)	9 (4.79)	18 (7.83)		
**Treatment stage**	CAR-T cell therapy	15 (3.09)	2 (2.94)	5 (2.66)	8 (3.48)	35.593^a^	< 0.001
	Maintenance	73 (15.02)	23 (33.82)	18 (9.57)	32 (13.91)		
	Post-transplantation	23 (4.73)	7 (10.29)	8 (4.26)	8 (3.48)		
	Before-transplantation	64 (13.17)	3 (4.41)	26 (13.83)	35 (15.22)		
	Post-chemotherapy	258 (53.09)	28 (41.18)	101 (53.72)	129 (56.09)		
	Before-chemotherapy	53 (10.91)	5 (7.35)	30 (15.96)	18 (7.83)		
**Relapse**	Yes	83 (17.08)	12 (17.65)	25 (13.30)	46 (20.00)	3.299^b^	0.192
	None	403 (82.92)	56 (82.35)	163 (86.70)	184 (80.00)		
**Insurance**	Social medical insurance (Provincial, municipal and urban medical insurance)	146 (30.04)	28 (42.18)	43 (22.87)	75 (32.61)	58.005^b^	< 0.001
	New rural Co-operative medical system	133 (27.37)	27 (39.71)	42 (22.34)	64 (27.83)		
	Commercial insurance/public medical insurance	147 (30.25)	11 (16.18)	56 (29.79)	80 (34.78)		
	Self-paying	60 (12.35)	2 (2.94)	47 (25.00)	11 (4.78)		
**Complication**	None	353 (72.63)	49 (72.06)	136 (72.34)	168 (73.04)	0.039^b^	0.981
	Yes	133 (27.37)	19 (27.94)	52 (27.66)	62 (26.96)		
**Sense of coherence**	Low	209 (43.00)	22 (32.35)	73 (38.83)	114 (49.57)	8.969^a^	0.063
	Medium	176 (36.21)	31 (45.59)	73 (38.83)	72 (31.30)		
	High	101 (20.78)	15 (22.06)	42 (22.34)	44 (19.13)		
**Social support**	Low	97 (19.96)	10 (14.71)	31 (16.49)	56 (24.35)	20.244^b^	< 0.001
	Medium	175 (36.01)	16 (23.53)	64 (34.04)	95 (41.30)		
	High	214 (44.03)	42 (61.76)	93 (49.47)	79 (34.35)		

HMs, hematologic malignancies; CAR-T, Chimeric Antigen Receptor T-cell. C1, No-financial toxicity group; C2, Mild financial toxicity group; C3, Moderate financial toxicity group.

a Fisher's Exact Test.

b Chi-square value.

### Financial toxicity and anxiety and depression

The mean financial toxicity score among patients was 17.25 ± 10.74, indicating varying degrees of financial burden. The average score for anxiety and depression, as measured by the PHQ-4, was 3.56 ± 2.97.

### Latent profile analysis (LPA)

**Fig. 1:**
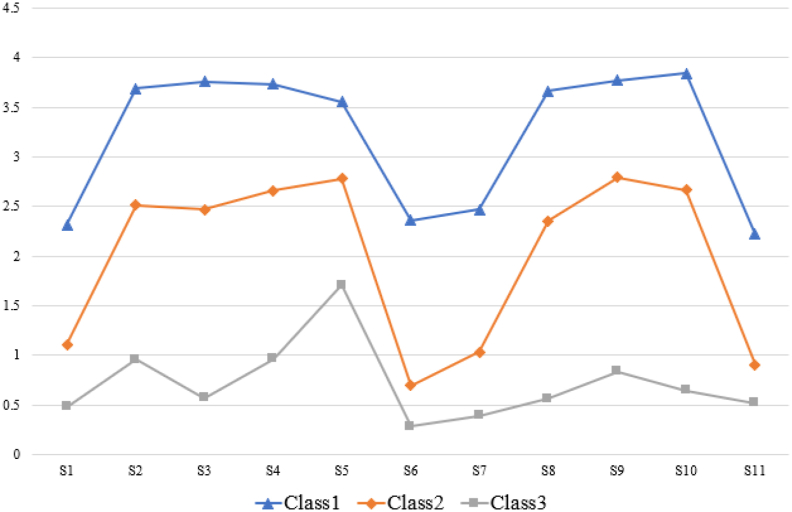
Latent profile analysis of financial toxicity in patients with hematological malignancies (Class 1, No-financial toxicity group; Class 2, Mild financial toxicity group; Class 3, Moderate financial toxicity group).

LPA was conducted using the 11 items of the COST scale as manifest indicators. Five models were tested, with fit indices presented in Table 2. As the number of latent classes increased, AIC, BIC, and adjusted BIC values decreased. The three-class model demonstrated the highest entropy value and significant LMR and BLRT values (*P* < 0.05), indicating optimal model fit. Therefore, the three-class model was selected as the best representation of the data. Based on financial toxicity scores, the classes were defined as follows: (1) Class 1 (C1, *n* = 68, 13.99%): No financial toxicity (mean score = 7.95). (2) Class 2 (C2, *n* = 188, 38.68%): Mild financial toxicity (mean score = 22.06). (3) Class 3 (C3, *n* = 230, 47.33%): Moderate financial toxicity (mean score = 35.43). Fig. 1 illustrates the distribution of financial toxicity scores across the three latent classes.

**Table 2:** Result of the latent profile analysis.

Class	AIC	BIC	aBIC	Entropy	LMR(*P*)	BLRT(*P*)	Proportions
1	17,851.54	17,943.64	17,873.81	–	–	–	–
2	15,573.58	15,715.91	15,608.00	0.930	0.000	0.000	0.55/0.45
3	14,912.74	15,105.31	14,959.31	0.936	0.002	0.000	0.47/0.14/0.39
4	14,591.92	14,834.72	14,650.63	0.893	0.238	0.000	0.30/0.33/0.25/0.11
5	14,337.48	14,630.51	14,408.33	0.913	0.217	0.000	0.06/0.28/0.27/0.28/0.11

AIC, Akaike Information Criterion; BIC, Bayesian Information Criterion; aBIC, adjusted Bayesian Information Criterion; LMRT, Lo-Mendell–Rubin Test; BLRT, Bootstrap Likelihood Ratio Test.

### General data of patients with HMs in the three categories between groups

Univariate analysis revealed statistically significant differences among the three financial toxicity classes in terms of age, sex, marital status, educational level, occupation, residence, family monthly income, diagnosis, chronic diseases, course of disease, treatment stage, insurance, and social support (Table 1).

**Table 3:** Influencing factors for HMs patients in different financial toxicity groups.

Reference group		C1						C2		
Comparison group		C2			C3			C3		
		OR	95% CI	*P*	OR	95% CI	*P*	OR	95% CI	*P*
**Sex**	Male	0.378	0.150–0.950	0.039	0.554	0.227–1.352	0.195	0.682	0.402–1.157	0.156
	Female	0^a^			0^a^			0^a^		
**Marital status**	Unmarried	18.616	0.703–492.732	0.080	3.913	1.661–1508.496	0.024	0.372	0.034–4.101	0.419
	Married	5.446	0.867–34.204	0.071	2.836	2.037–142.512	0.009	0.320	0.038–2.671	0.292
	Divorced	35.109	1.510–816.258	0.027	4.661	3.760–2974.959	0.006	0.332	0.028–3.876	0.379
	Widowed	0^a^			0^a^			0^a^		
**Occupation**	Farmer/Worker	3.968	1.057–14.899	0.041	3.129	0.847–11.551	0.087	1.268	0.581–2.767	0.550
	Eenterprise personnnel	5.816	1.368–24.722	0.017	2.537	0.619–10.405	0.196	2.292	0.994–5.288	0.052
	Civil servant/public institution	0.997	0.239–4.164	0.996	0.832	0.241–2.873	0.771	1.199	0.356–4.032	0.770
	Individual/Retired/Unemployed	0^a^			0^a^			0^a^		
**Residence**	Rural	2.643	0.686–10.184	0.158	1.151	0.304–4.364	0.836	2.295	1.052–5.009	0.037
	Township	3.815	1.080–13.481	0.038	3.024	0.889–10.288	0.076	1.262	0.571–2.787	0.565
	City	0^a^			0^a^			0^a^		
**Family monthly income (Yuan)**	> 10,000	29.734	5.245–168.564	0.000	9.850	1.753–55.352	0.000	3.019	1.341–6.795	0.008
	5000–10,000	4.715	1.785–12.455	0.002	3.004	1.196–7.543	0.002	1.570	0.808–3.051	0.184
	< 5000	0^a^			0^a^			0^a^		
**Diagnosis**	Multiple myeloma	0.768	0.048–12.216	0.852	0.658	0.048–9.018	0.754	1.167	0.299–4.562	0.824
	Lymphoma	1.115	0.065–19.160	0.940	0.201	0.013–3.030	0.246	5.556	1.347–22.920	0.018
	Leukemia	0.737	0.045–11.940	0.830	0.196	0.014–2.821	0.231	3.756	0.949–14.864	0.059
	Others	0^a^			0^a^			0^a^		
**Chronic disease**	None	2.152	0.771–6.005	0.143	0.542	0.210–1.396	0.205	3.970	2.170–7.263	0.000
	Yes (Hypertension, diabetes, coronary heart disease, chronic obstructive pulmonary disease)	0^a^			0^a^			0^a^		
**Course of disease**	1–6 months	0.260	0.053–1.265	0.095	1.089	0.234–5.071	0.913	0.239	0.075–0.758	0.015
	6–12 months	1.022	0.167–6.270	0.981	1.842	0.315–10.790	0.498	0.555	0.164–1.875	0.343
	1–3 years	2.017	0.337–12.089	0.443	1.336	0.219–8.144	0.753	1.509	0.443–5.136	0.510
	3+ years	0^a^			0^a^			0^a^		
**Treatment stage**	CAR-T cell therapy	0.176	0.010–3.202	0.240	0.168	0.011–2.482	0.194	1.044	0.199–5.467	0.959
	Maintenance	0.141	0.022–0.921	0.041	0.062	0.011–0.362	0.002	2.286	0.755–6.922	0.144
	Post-transplantation	0.213	0.020–2.276	0.201	0.193	0.021–1.802	0.149	1.105	0.269–4.529	0.890
	Before-transplantation	1.085	0.120–9.804	0.942	0.695	0.085–5.669	0.734	1.561	0.531–4.590	0.418
	Post-chemotherapy	0.864	0.153–4.885	0.868	0.473	0.094–2.366	0.362	1.827	0.756–4.414	0.181
	Before-chemotherapy	0^a^			0^a^			0^a^		
**Insurance**	Social medical insurance	0.291	0.044–1.942	0.202	0.048	0.008–0.286	0.001	6.087	2.391–15.496	0.000
	New rural Co-operative medical system	0.271	0.040–1.824	0.179	0.045	0.007–0.267	0.001	6.051	2.333–15.698	0.000
	Commercial insurance/public medical insurance	0.749	0.107–5.230	0.771	0.128	0.021–0.799	0.028	5.832	2.300–14.791	0.000
	Self-paying	0^a^			0^a^			0^a^		
**Social support**	Low	3.056	0.917–10.190	0.069	1.487	0.454–4.868	0.512	2.055	1.013–4.170	0.046
	Medium	3.104	1.172–8.226	0.023	2.122	0.839–5.362	0.112	1.463	0.822–2.604	0.196
	High	0^a^			0^a^			0^a^		

HMs, hematologic malignancies; CAR-T, Chimeric Antigen Receptor T-cell; OR, odds ratio; CI, Confidence Interval.

C1, No-financial toxicity group; C2, Mild financial toxicity group; C3, Moderate financial toxicity group.

a Reference group.

### Influencing factors for HMs patients in different financial toxicity groups

Multinomial Logistic regression was conducted using variables that were statistically significant in univariate analysis. There was no evidence of multicollinearity (VIF < 5). Using the “no financial toxicity” profile as the reference, sex, marital status, occupation, residence, family monthly income, treatment stage, insurance, and social support were found to be significant predictors of membership in either the mild or moderate financial toxicity profiles.

Subsequently, when the mild financial toxicity profile was used as the reference, residence, family monthly income, diagnosis, chronic disease, course of disease, insurance, and social support were significant predictors of membership in the moderate financial toxicity profile. Detailed regression results are presented in Table 3.

### Comparison of anxiety and depression scores across financial toxicity profiles

Statistically significant differences were observed in anxiety and depression scores among the three latent financial toxicity profiles of patients with HMs (*P* < 0.05). Posterior multiple comparisons revealed a progressive increase in anxiety and depression scores corresponding to higher levels of financial toxicity. Specifically, patients in the moderate financial toxicity profile exhibited significantly higher psychological distress compared to those in the mild or no financial toxicity profiles. Detailed results are presented in Table 4.

**Table 4:** Comparison of Anxiety-Depression scores with financial toxicity.

Variable	Class			Kruskal–Wallis test	*P*	Posterior comparisons
	C1	C2	C3			
Anxiety-depression score (mean ± SD)	1.99 ​± ​2.82	3.01 ​± ​2.48	4.48 ​± ​3.08	55.36	< 0.001	C3 > C2 >C1

SD, standard deviation; C1, No-financial toxicity group; C2, Mild financial toxicity group; C3, Moderate financial toxicity group.

## Discussion

### Main findings

This study identified a mean financial toxicity score of 17.25 ± 10.74 among patients with HMs, indicating a moderate level of financial burden as measured by the COST. This score is higher than that reported by Wang et al.^12^ for a similar population, but consistent with findings from Zhang et al.^24^ and Shao et al.^10^ in patients with lung and prostate cancers, respectively. Several factors may contribute to this elevated financial burden.

First, the nature of HMs often necessitates long-term, high-cost, and urgent treatments, such as CAR-T therapy, which can cost several million yuan per course.^25^ Additionally, aggressive subtypes, such as acute leukemias, demand immediate initiation of intensive therapy, placing acute financial stress on families. Second, although some earlier studies were conducted approximately two years ago, since then, newer therapies, particularly targeted agents and immunotherapies, have emerged but are not yet widely reimbursed by health insurance, leading to substantial out-of-pocket expenditures for patients.

These findings highlight the urgent need for improved health policy measures, enhanced financial support systems, and integrated clinical management approaches to reduce financial toxicity in this population.

Regarding psychological distress, the mean Patient Health Questionnaire-4 (PHQ-4) score was 3.56 ± 2.97, reflecting a mild overall level of anxiety and depression. However, 62.14% of patients scored ≥ 3, exceeding the clinical threshold for psychological concern. This prevalence is notably higher than that reported among patients with solid tumors,^26^ likely due to the distinct psychological stressors associated with HMs. Unlike many solid tumors,^27^ which often follow more structured treatment pathways, HMs are characterized by complex, evolving regimens and prolonged treatment durations, which contribute to persistent uncertainty. Furthermore, patients with HMs face a higher risk of severe treatment-related complications (e.g., myelosuppression, infections), frequent relapses, and comparatively poorer prognoses, all of which may exacerbate emotional distress.

These findings underscore the necessity of integrating routine psychological screening and mental health support into the standard care protocols for patients with HMs.

LPA classified the financial toxicity of patients with HMs into three distinct groups: non-financial toxicity (13.99%), mild financial toxicity (38.68%), and moderate financial toxicity (47.33%). Unlike the high/medium/low profiles identified in young and middle-aged pan-cancer studies^13^^,^^14^ and the mild/moderate resource-deficient/ moderate balanced/severe profiles in lung cancer patients,^24^ this study did not detect a high financial toxicity group. Several contextual and sample-specific factors may explain this difference. For example, Lin's study focused on young and middle-aged cancer patients, a demographic that often bears primary family responsibilities and faces considerable economic burden. In fact, 66.4% of Lin's sample and 74.4% of Su's sample reported a family monthly income below 5000 yuan,^13^^,^^14^ while only 32.72% of participants in our study reported a monthly household income below this threshold, suggesting a potentially lower level of economic vulnerability. Similarly, Zhang's study targeted lung cancer patients, whose disease is characterized by pathological heterogeneity,^24^ often requiring prolonged, complex, and costly treatment regimens,^28^ thereby contributing to more severe financial distress. In comparison, relatively fewer patients in our study underwent high-cost therapies such as CAR-T (3.09%), which may have contributed to the absence of a high financial toxicity profile. The medium financial toxicity group may exhibit severe treatment affordability challenges, diminished quality of life, and heightened psychological distress, warranting prioritized clinical interventions. Notably, the low and non-toxicity groups, though currently less affected, represent a high-risk transitional population susceptible to progression as treatment expenses accumulate, highlighting the need for regular financial screening and dynamic monitoring to enable early support and mitigate toxicity escalation. These findings underscore the unique economic burden profile of HMs and emphasize the importance of context-specific assessment and preemptive management strategies.

In the comparison between mild financial toxicity and no financial toxicity, the Logistic analysis of this study found that HMs at highest risk for mild financial toxicity were those with: female,^29^ divorced,^30^ farmer/worker or enterprise employment,^9^^,^^31^ township residence,^9^ lower family income,^9^^,^^16^^,^^32^ non-treatment maintenance period,^10^^,^^31^ and moderate social support,^33^ which was consistent with previous studies. Sex disparities emerged clearly, with female patients facing greater toxicity due to caregiving-related income loss and persistent wage gaps.^29^ Marital status significantly influenced outcomes, as divorced/widowed patients are more likely to fall into economic hardship due to lacked crucial spousal support.^30^ Regarding careers, employment in enterprises posed particular risk due to treatment-related job instability and subsequent earning reductions,^34^ compounded by reliance on family savings for medical costs, similar to the research results of Li et al.^9^^,^^31^ Additionally, farmers and workers often lack stable income sources, they may experience delays in diagnosis and treatment due to work constraints, all of which increase their vulnerability to financial toxicity.^35^ However, the classification of occupation in this study was not clearly defined, which may have introduced some degree of bias into the results. Geographic and socioeconomic factors played key roles. Township patients faced dual challenges of limited healthcare access and lower insurance coverage, while lower-income households experienced disproportionately higher burdens (5.86 × greater than high-income patients).^16^^,^^31^ The profound impact of treatment options on the financial toxicity experienced by patients should not be underestimated. Shao et al. 's^10^ research on the financial toxicity of colorectal cancer patients shows that the postoperative duration is related to financial toxicity. However, the results of this study indicate that the risk of patients in the non-treatment maintenance period is increased, which may be related to the decrease in the medical insurance reimbursement ratio at this stage, but the continuous generation of follow-up and rehabilitation costs. Regarding social support, the research results differ from those of Li et al.^9^ In this study, the financial toxicity of moderate social support was high.^33^ This might be because, compared with the low support group, patients in the moderate support group might neglect seeking help due to the “barely maintaining” state, while compared with the high support group, the economic assistance they received was significantly insufficient, this effect makes them susceptible to financial toxicity.^9^

In the comparison between moderate financial toxicity and no-financial toxicity, patients with HMs demonstrating moderate financial toxicity were more likely to be non-widowed (unmarried, married, or divorced),^29^^,^^30^ have a monthly income above 5000 yuan,^16^ be outside the treatment maintenance phase,^10^ or lack medical insurance.^11^^,^^31^ Non-widowed patients may experience heightened financial strain due to variable family support systems, while married individuals often benefit from spousal assistance, unmarried or divorced patients typically lack stable economic support.^30^^,^^34^ Lower income^9^ and active treatment (non-maintenance)^10^ correlated with moderate financial toxicity, reflecting increased treatment-related expenses. Chinese insurance system is a social security mechanism that provides financial support to retirees through mandatory or voluntary contributions.^31^ The risk characteristics of patients without medical insurance are particularly prominent.^11^^,^^31^ Against the gradual improvement of the Chinese multi-level medical security system, such patients often face more severe economic predicaments.

In the comparison between the mild and moderate financial toxicity groups, we found that patients in the moderate financial toxicity group were more likely to reside in rural areas,^9^ family earn > 10,000 yuan monthly,^9^^,^^16^^,^^34^ have lymphoma diagnoses,^10^ lack chronic comorbidities,^10^ exhibit shorter disease durations,^31^ possess insurance coverage,^31^ and demonstrate low social support.^9^ Rural residents may experience heightened financial strain due to limited healthcare access, elevated transportation costs, and constrained incomes. Lymphoma patients face particularly severe financial toxicity given the disease's complex treatment regimens and associated costs. Those with shorter disease durations encounter acute economic pressures from substantial upfront diagnostic and treatment expenses. Notably, insured patients and those without chronic conditions showed unexpectedly higher financial toxicity rates, contrary to findings by Li et al.^9^ and Li et al.^31^ General data show that the proportion of patients with chronic diseases in the moderate economic toxicity group is the highest (42.02%), patients with chronic diseases usually have adapted to long-term medical expenses or have been included in the medical insurance chronic disease reimbursement policy.^11^ In contrast, those without chronic diseases may lack experience in dealing with sudden high medical expenses, resulting in more obvious financial toxicity. The results in terms of insurance show that there are differences between the non-economically toxic and mild to moderate groups, the statement “having insurance” does not necessarily mean “low financial stress”. The economically toxic group with insurance is relatively higher. The analysis of the reasons may be related to the relative economic pressure caused by insufficient insurance coverage or differences in treatment options for patients, although they have insurance.

This study reveals a significant positive correlation between financial toxicity and symptoms of anxiety and depression among patients with HMs, consistent with findings from Yu^19^ in adult cancer populations with all types of cancer. HMs often require prolonged, high-cost treatments such as stem cell transplantation, targeted therapy, and immunotherapy.^19^^,^^20^ Relapse and drug resistance may further elevate expenses, and over time, both direct (medications, hospitalization) and indirect costs (lost income, caregiving) accumulate, gradually eroding household finances and patients’ sense of control.^18^ This persistent financial stress may act as a chronic psychological burden, contributing to anxiety and depression.^26^ Additionally, HMs frequently affect individuals in early or middle adulthood. In this study, 41% of participants were aged 40–59, a group often serving as primary family breadwinners. From the moment of diagnosis, patients may stop working and begin to rely on family members for care. This increased dependence can evoke feelings of guilt,^36^ stigma,^37^ and emotional distress,^38^^,^^39^ along with reluctance to express personal needs,^38^ which are further intensified by financial strain, thereby increasing vulnerability to depressive symptoms. Lee^40^ highlighted the stage-specific nature of financial toxicity. While patients may focus on survival early in treatment, financial concerns often intensify later, as ongoing costs and uncertainty about future economic security take a toll.^27^ These findings underscore that the mental health impact of financial toxicity is both progressive and cumulative, especially in the later stages of prolonged treatment, aligning with that higher levels of financial toxicity are associated with greater symptoms of anxiety and depression among patients with HMs.

### Implications for nursing practice and research

The findings of this study highlight the need for stratified management strategies in nursing practice to address financial toxicity, particularly among high-risk subgroups. Tailored interventions should be developed for female patients^20^ and those residing in rural or township areas,^3^ with a focus on improving employment protections and expanding access to localized healthcare services.^20^ For divorced individuals, personalized financial support including guidance on applying for charitable assistance and integrated psychosocial services^24^ are essential to mitigate both economic and emotional burdens.

System-level reforms are also warranted. Policies such as cross-regional, real-time health insurance reimbursement should be implemented to ensure greater flexibility in coverage regardless of treatment location. For farmers, workers, and enterprise employees, adjusting reimbursement caps and offering tailored medical loan programs may help reduce out-of-pocket costs. Given the exorbitant cost of certain therapies, such as CAR-T, supplementary insurance coverage should be mandated to promote equitable access.

For patients undergoing intensive treatments, flexible employment policies should be encouraged to support continued workforce participation and financial stability.^3^ A tripartite support framework involving government, civil society, and charitable organizations is needed, including automatic referral mechanisms to social workers for low-income patients, facilitating timely access to public welfare programs.^19^ For patients not currently undergoing active treatment, subsidies for transportation and nutritional support provided in partnership with non-profit organizations can help reduce indirect economic burdens.^19^

Additionally, financial counseling interventions should be initiated before the maintenance phase of treatment to proactively manage emerging financial toxicity.^17^^,^^19^ Routine screening for socioeconomic vulnerability is recommended, particularly for individuals with limited social support. Early identification of financial strain allows for timely intervention and may prevent long-term socioeconomic decline.^17^ These multifaceted approaches should be further explored through future research to establish evidence-based nursing models for managing financial toxicity among patients with HMs.

### Limitations

This study has several limitations that should be acknowledged. First, as a cross-sectional design, causal relationships cannot be established, nor can dynamic changes in financial toxicity and psychological distress over time be captured. For instance, patients who self-fund their treatment may opt for less expensive therapies due to pre-existing financial constraints, rather than insurance coverage directly influencing financial toxicity. Second, some potential confounding variables were not included in the analysis. The omission of key factors such as household debt and non-medical expenses may have led to an overestimation of the impact of treatment-related costs on financial toxicity. Third, the use of convenience sampling in provincial hospitals may introduce selection bias, potentially limiting the generalizability of findings to patients from less-developed or rural regions. Fourth, data collection relied on self-reported questionnaires, which are susceptible to recall bias and may affect data accuracy. Additionally, 23 questionnaires were completed by researchers on behalf of participants due to literacy or comprehension limitations, introducing potential response bias. Lastly, certain classification ambiguities during data collection, such as the categorization of age groups and occupations may have introduced bias, potentially affecting the precision of analyses. Future research should employ randomized sampling strategies across multiple hospital levels to enhance representativeness. Integrating self-reported data with objective measures and conducting longitudinal studies would allow for a more robust understanding of temporal changes in financial toxicity and its psychological and behavioral sequelae. Expanding the range of variables collected will further elucidate the complex mechanisms underlying financial toxicity in patients with HMs.

## Conclusions

Patients with HMs experience heterogeneous levels of financial toxicity. Through LPA, this study identified three distinct profiles: no financial toxicity, mild financial toxicity, and moderate financial toxicity. Key factors influencing financial toxicity included sex, marital status, occupation, residence, family monthly income, diagnosis, chronic disease, treatment stage, insurance, and social support. To effectively mitigate financial toxicity, healthcare providers should develop tailored interventions aligned with patients’ socioeconomic profiles to promote optimal treatment adherence and improve recovery outcomes. However, this study was limited to inpatients at provincial-level hospitals, which may limit the generalizability of findings. Future research should broaden the scope to include outpatients and patients from varied healthcare settings, such as county- and municipal-level hospitals and incorporate stratified analyses based on urban-rural differences and insurance categories to enhance the precision of financial toxicity management strategies.

## CRediT authorship contribution statement

**Guiyuan Ma and Nannan Long:** Conceptualization, Methodology, Performed the data analysis, Writing –Original draft. **Chengyuan Li:** Project Administration, Funding Acquisition, Writing – Reviewing and Supervision. **Ping Mao:** Validation. **Yuanyuan Li and Fang Li:** Data collection. All authors have read and approved the final manuscript.

## Ethics statement

This study was approved by the Ethics Committee of the third Xiangya hospital, Central South University (Approval No. 2024-S425). Written informed consent was obtained from all participants before data collection, and the clinical data and basic information of all participants were strictly confidential. All procedures complied with the 1964 Helsinki Declaration and its later amendments or comparable ethical standards.

## Data availability statement

The data that support the findings of this study are available from the corresponding author upon reasonable request.

## Declaration of generative AI and AI-assisted technologies in the writing process

No AI tools/services were used during the preparation of this work.

## Funding

This work was supported by the 10.13039/501100004735Hunan Natural Science Foundation (Grant Nos. 2024JJ9236 and 2025JJ60535), Hunan Graduate Research Innovation Project (Grant No. CX20240325). The funders had no role in considering the study design or in the collection, analysis, interpretation of data, writing of the report, or decision to submit the article for publication.

## Declaration of competing interest

The authors declare no conflict of interest.

## References

[1] Tseng Y.D., Ng A.K., Malignancies Hematologic (2020). Hematol Oncol Clin N Am.

[2] Bray F., Laversanne M., Sung H. (2024). Global cancer statistics 2022: GLOBOCAN estimates of incidence and mortality worldwide for 36 cancers in 185 countries. CA Cancer J Clin.

[3] Zhang N., Wu J., Wang Q. (2023). Global burden of hematologic malignancies and evolution patterns over the past 30 years. Blood Cancer J.

[4] Bonafede M., Anaissie E., Evans K. (2021). Healthcare utilization and cost of cancer-related care prior to allogeneic hematopoietic cell transplantation for hematologic malignancies in the US: a retrospective real-world analysis. BMC Health Serv Res.

[5] Dabas P., Danda A. (2023). Revolutionizing cancer treatment: a comprehensive review of CAR-T cell therapy. Med Oncol.

[6] Abrams H.R., Durbin S., Huang C.X. (2021). Financial toxicity in cancer care: origins, impact, and solutions. Transl Behav Med.

[7] de Souza J.A., Yap B.J., Wroblewski K. (2017). Measuring financial toxicity as a clinically relevant patient-reported outcome: the validation of the COmprehensive Score for financial Toxicity (COST). Cancer.

[8] Yu H.H., Bi X., Liu Y.Y. (2017). [Reliability and validity of the Chinese version on Comprehensive Scores for Financial Toxicity based on the patient-reported outcome measures]. Zhonghua Liuxingbingxue Zazhi.

[9] Li T., Cui P., Shao M. (2024). Financial toxicity and its influencing factors in patients with non-Hodgkin lymphoma: a cross-sectional study. Eur J Oncol Nurs.

[10] Shao M., Yao L., Zhang M. (2024). Post-surgery financial toxicity and its influencing factors in colorectal cancer care: a cross-sectional study. Eur J Oncol Nurs.

[11] Ng A.P., Sanaiha Y., Verma A. (2022). Insurance-based disparities and risk of financial toxicity among patients undergoing gynecologic cancer operations. Gynecol Oncol.

[12] Wang Y., Luo B., Wang X. (2024). Financial toxicity, coping strategies, and quality of life among Chinese patients with hematologic malignancies: a cross-sectional study. Support Care Cancer.

[13] Su M., Liu S., Liu L. (2023). Heterogeneity of financial toxicity and associated risk factors for older cancer survivors in China. iScience.

[14] Lin X., Teng X., Lan J. (2025). Financial toxicity and its influencing factors in young and middle-aged cancer patients: a latent profile analysis. Support Care Cancer.

[15] Myers S.P., Aviki E., Sevilimedu V. (2024). Financial toxicity among women with breast cancer varies by age and race. Ann Surg Oncol.

[16] Xiao T., Zhong H., Xiao R. (2024). Profiles of financial toxicity and influencing factors among cancer patients: a latent profile analysis. Res Soc Adm Pharm.

[17] Xu B., So W.K.W., Choi K.C. (2024). Financial toxicity and its risk factors among patients with cancer in China: a nationwide multisite study. Asia Pac J Oncol Nurs.

[18] Carrera P.M., Kantarjian H.M., Blinder V.S. (2018). The financial burden and distress of patients with cancer: understanding and stepping-up action on the financial toxicity of cancer treatment. CA Cancer J Clin.

[19] Yu H., Li H., Zuo T. (2022). Financial toxicity and psychological distress in adults with cancer: a treatment-based analysis. Asia Pac J Oncol Nurs.

[20] Yücel K.B., Özay Z.I., Sütcüoğlu O. (2023). Greater financial toxicity correlates with increased psychological distress and lower quality of life among Turkish cancer patients. Support Care Cancer.

[21] Nylund-Gibson K., Garber A.C., Carter D.B. (2023). Ten frequently asked questions about latent transition analysis. Psychol Methods.

[22] Löwe B., Wahl I., Rose M. (2010). A 4-item measure of depression and anxiety: validation and standardization of the Patient Health Questionnaire-4 (PHQ-4) in the general population. J Affect Disord.

[23] Zimet G.D., Powell S.S., Farley G.K. (1990). Psychometric characteristics of the multidimensional scale of perceived social support. J Pers Assess.

[24] Zhang X., Zhang L., Geng Z. (2025). Potential profile analysis of financial toxicity and its related factors among lung cancer patients. BMC Cancer.

[25] Kc M., Oral E., Rung A.L. (2023). Prostate cancer aggressiveness and financial toxicity among prostate cancer patients. Prostate.

[26] Abuelgasim K.A., Ahmed G.Y., Alqahtani J.A. (2016). Depression and anxiety in patients with hematological malignancies, prevalence, and associated factors. Saudi Med J.

[27] Zeilinger E.L., Oppenauer C., Knefel M. (2022). Prevalence of anxiety and depression in people with different types of cancer or haematologic malignancies: a cross-sectional study. Epidemiol Psychiatr Sci.

[28] Nasim F., Sabath B.F., Eapen G.A. (2019). Lung cancer. Med Clin.

[29] De Felice F., Locati L.D., Ronchi S. (2022). Quality of life and financial toxicity after (chemo)radiation therapy in head and neck cancer: are there any sex- or sex-related differences?. Tumori.

[30] Desai A., Jella T.K., Cwalina T.B. (2022). Demographic analysis of financial hardships faced by brain tumor survivors. World Neurosurg.

[31] Li F., Xiao T., Liu C. (2025). Explore potential profiles and influencing factors for financial toxicity in patients with colorectal cancer undergoing chemotherapy: a cross-sectional study. Semin Oncol Nurs.

[32] Ehsan A.N., Wu C.A., Minasian A. (2023). Financial toxicity among patients with breast cancer worldwide: a systematic review and meta-analysis. JAMA Netw Open.

[33] XiuCen W., GuiHua C., Qin L. (2024). Factors influencing economic toxicity and coping strategies in lung cancer patients: a scoping review. Heliyon.

[34] Mols F., Tomalin B., Pearce A. (2020). Financial toxicity and employment status in cancer survivors. A systematic literature review. Support Care Cancer.

[35] Hassen A.M., Hussien F.M., Asfaw Z.A. (2021). Factors associated with delay in breast cancer presentation at the only oncology center in north East Ethiopia: a cross-sectional study. J Multidiscip Healthc.

[36] Haozous E.A., Knobf M.T., Brant J.M. (2011). Understanding the cancer pain experience in American Indians of the Northern Plains. Psychooncology.

[37] Shi D., Li R., Chen P. (2024). The mediating effect of stigma on the relationship between fear of disease progression and social alienation in patients with haematological malignancies. Hematology.

[38] Serçe Ö., Günüşen N.P. (2021). The interaction between hematological cancer patients and family caregivers and their life changes: a qualitative dyadic approach. Cancer Nurs.

[39] Monterosso L., Taylor K., Platt V. (2017). A qualitative study of the post-treatment experiences and support needs of survivors of lymphoma. Eur J Oncol Nurs.

[40] Lee S., Olvera R.G., Shiu-Yee K. (2023). Short-term and long-term financial toxicity from breast cancer treatment: a qualitative study. Support Care Cancer.

